# Photoluminescent Scaffolds Based on Natural and Synthetic Biodegradable Polymers for Bioimaging and Tissue Engineering

**DOI:** 10.3390/life13040870

**Published:** 2023-03-24

**Authors:** Ekaterina M. Trifanova, Gulalek Babayeva, Maria A. Khvorostina, Aleksandra V. Atanova, Maria E. Nikolaeva, Anastasia V. Sochilina, Evgeny V. Khaydukov, Vladimir K. Popov

**Affiliations:** 1 Federal Scientific Research Centre “Crystallography and Photonics” of Russian Academy of Sciences, 119333 Moscow, Russia; 2 N.N. Blokhin National Medical Research Center of Oncology, Ministry of Health of Russia, 115478 Moscow, Russia; 3 Research Institute of Molecular and Cellular Medicine, RUDN University, 117198 Moscow, Russia; 4 Research Centre for Medical Genetics, 115478 Moscow, Russia; 5 Institute of Physics, Technology, and Informational Systems, Moscow State Pedagogical University, 119991 Moscow, Russia; 6 Shemyakin & Ovchinnikov Institute of Bioorganic Chemistry of Russian Academy of Sciences, 117997 Moscow, Russia

**Keywords:** collagen, PLGA, hyaluronic acid, electrospinning, 3D printing, upconversion nanoparticles, bioimaging, scaffold implantation

## Abstract

Non-invasive visualization and monitoring of tissue-engineered structures in a living organism is a challenge. One possible solution to this problem is to use upconversion nanoparticles (UCNPs) as photoluminescent nanomarkers in scaffolds. We synthesized and studied scaffolds based on natural (collagen—COL and hyaluronic acid—HA) and synthetic (polylactic-co-glycolic acids—PLGA) polymers loaded with β-NaYF_4_:Yb^3+^, Er^3+^ nanocrystals (21 ± 6 nm). Histomorphological analysis of tissue response to subcutaneous implantation of the polymer scaffolds in BALB/c mice was performed. The inflammatory response of the surrounding tissues was found to be weak for scaffolds based on HA and PLGA and moderate for COL scaffolds. An epi-luminescent imaging system with 975 nm laser excitation was used for in vivo visualization and photoluminescent analysis of implanted scaffolds. We demonstrated that the UCNPs’ photoluminescent signal monotonously decreased in all the examined scaffolds, indicating their gradual biodegradation followed by the release of photoluminescent nanoparticles into the surrounding tissues. In general, the data obtained from the photoluminescent analysis correlated satisfactorily with the histomorphological analysis.

## 1. Introduction

Modern biomaterials and cellular technologies make it possible to create tissue-engineered constructs (TECs) known to achieve successful replacement of tissue defects in human and animal bodies, as well as targeted regeneration of certain tissue types [1]. As a rule, such TECs contain three-dimensional biocompatible scaffolds that act as a volumetric structure and provide the vital activity of various cell cultures [2,3]. Designing multimodal TECs and studying their functional properties in vivo is a promising direction in modern regenerative medicine [4]. The first main step in developing a TEC is to choose manufacturing technics and materials matching the selected purpose. Materials and architectonics of such TECs should enable not only effective adhesion, differentiation, and proliferation of cell cultures, but also bioimaging and monitoring of the processes taking place in real time [5,6]. Ideally, the constructs should additionally carry out targeted and prolonged delivery of various bioactive components (growth factors, drugs, etc.) to the implantation area [7,8]. These bioactive components can be added at different stages of scaffold manufacturing. After a finished scaffold is obtained it can be incubated with certain types of cells and then implanted into an organism for further defect repair.

Currently, various luminescent nanomarkers are actively used to visualize living cells and their organelles in various in vitro studies [9,10]. However, the possibility of bioimaging the TEC and the state of the surrounding living tissues in vivo is of much greater interest. Several different bioimaging methods such as fluorescence analysis [11], computed [12] and magnetic resonance tomography [13], Raman spectroscopy [14], dark-field microscopy [15], two-photon fluorescence [16], and photoacoustic imaging [17] are widely used. The techniques based on photoluminescence have high spatial resolution, are intuitive for users, and are relatively inexpensive.

One of the main barriers to the development of TECs with bioimaging systems is that although there are multiple available nanoprobes, many of them are limited in versatility and/or biocompatibility. Photoluminescent nanomarkers for optical imaging should be non-toxic, hydrophilic, and able to persist in the body for long-term observation [18,19]. The photoluminescent properties of nanoparticles must be sensitive to the microenvironment in order to adequately reflect the state of the biological tissues [20]. In addition, the photoluminescence spectrum and the excitation line of nanomarkers should be within the optical “transparency window” of the biological tissues [21].

Luminescent nanomarkers for in vivo imaging fall into two groups: organic [22] and inorganic [23]. Organic luminescent nanomarkers are highly biocompatible but are less stable and have worse characteristics of quantum yield and luminescence lifetime than inorganic ones [24]. The most common inorganic nanomarkers are gold nanoparticles [25], graphene-based nanomaterials [26], quantum dots [27], and upconversion nanoparticles (UCNPs) [28]. It should be noted that the toxicity of most inorganic nanomarkers is still under discussion [29,30].

The application of lanthanide-based UCNPs helps to avoid many problems associated with conventional imaging probes, and provides visible or near-infrared emission under excitation at the 970–980 nm wavelength [31]. The UCNPs perform the sequential absorption of several photons through the long lifetime and ladder-like energy levels of trivalent lanthanide ions embedded in an inorganic host matrix. The emission spectral band corresponds to the so-called “transparency window” (650 to 1300 nm) of biological tissue, in which light penetration into the tissue occurs with minimal absorption and scattering [21]. The most efficient host matrix of UCNPs is NaYF_4_ co-doped with Yb^3+^ as a sensitizer and Er^3+^ or Tm^3+^ as an emitter. These UCNPs demonstrate unique upconversion emission with narrow emission lines, large anti-Stokes shift of several hundred nanometers, non-photoblinking, and superior photostability [32]. The toxicity of UCNPs is fairly well studied and depends on their size, concentration and surface modification protocols [33]. Moreover, UCNPs can be covered with different shells to embrace and alter their properties for certain purposes (for example, covering them with bioinert and hydrophilic shells [34]). All the properties of UCNPs listed above make them very attractive not only for bioimaging of tissue engineering constructs but also for treatment and drug delivery [35]. Using UCNPs as photoluminescent nanomarkers is useful for multifunctional bioimaging and the possibility of simultaneous drug delivery.

This study presents an approach to the design of scaffolds impregnated with as-synthesized hydrophobic UCNPs for in vivo analysis of the tissue response of laboratory animals using intravital visualization. Based on histomorphological analysis, we demonstrated that UCNP photoluminescent signals indicate scaffold biodegradation. Furthermore, we studied the inflammatory response of the surrounding tissues to HA, PLGA, and COL scaffold implantation.

## 2. Materials and Methods

### 2.1. Materials

As initial compounds for the formation of scaffolds, we used type I collagen (COL, Nearmedic Plus LLC, Moscow, Russia) polylactic-co-glycolic acid (PLGA, Purasorb PDLG7507 Corbion PURAC Biochem, Gorinchem, The Netherlands) with an inherent viscosity midpoint of 0.7 dL/g and a lactic-to-glycolic-acid monomer ratio of 75:25, and hyaluronic acid modified with glycidyl methacrylate (HAGM, synthesized at the Federal Research Center “Crystallography and Photonics” of the Russian Academy of Sciences according to the method described in [36]). The scaffolds were prepared by electrospinning and antisolvent and extrusion 3D printing followed by photocuring [20], briefly described below. As solvents, 1,1,1,3,3,3-hexafluoroisopropanol (HFIP, 99%, P&M-Invest, Moscow, Russia) and tetraglycol (Sigma Aldrich, St. Louis, MO, USA) were used for scaffold fabrication. As a cross-linking agent, 1,4-butanediol diglycidyl ether (BDDGE, ≥95%, Sigma Aldrich, St. Louis, MO, USA) was used, and isopropanol (99%, Ekos-1, Moscow, Russia) was used for collagen chemical stabilization. Polyethylene glycol diacrylate (PEGDA, Sigma Aldrich, St. Louis, MO, USA), (Pharmstandard, Moscow, Russia), and triethanolamine (Sigma-Aldrich, St. Louis, MO, USA) were used to provide photocuring of the HAGM. Reagents for UCNP synthesis (Y_2_O_3_, Yb_2_O_3_, Er_2_O_3_, CF_3_COOH, (CF_3_COO)Na, 1-octadecene, and oleic acid) were purchased from Sigma Aldrich, St. Louis, MO, USA.

### 2.2. Synthesis of Upconversion Nanoparticles

The lanthanide-doped NaYF_4_ nanocrystals were synthesized via the coordinate stabilization of yttrium, ytterbium, and erbium metal salts in a solution of oleic acid and octadecene, carried out by heating at a rate of 200 °C min^−1^ up to 320 °C in a Wood’s alloy bath in an oxygen-free atmosphere. The details of UCNP synthesis are described in detail elsewhere [37]. The core, formed from a nanocrystal of β-NaYF_4_ co-doped with the Yb^3+^ and Er^3+^ ions in 18% and 2% molar ratios, respectively, was coated with an undoped crystal shell of NaYF_4_, resulting in a core-shell structure of β-NaYF_4_:Yb^3+^, Er^3+^/NaYF_4_. The synthesis products were washed three times with 100% ethanol, and the nanoparticles were kept in a sealed container with 2-propanol. The synthesized nanoparticles were monodisperse, with an average diameter of ~21 ± 6 nm, and formed stable colloids in non-polar organic solvents such as hexane, chloroform, etc. The UCNPs were added into all the initial polymer solutions listed below to achieve a 1 wt.% (1 mg per 100 mg of polymer) concentration. All the initial polymer solutions were stirred in an ultrasound bath for 60 min for uniform distribution of nanoparticles in the composition.

### 2.3. Electrospinning

PLGA and COL solutions for electrospinning were prepared by dissolving polymers in HFIP to final concentrations of 9 wt.% and 4 wt.%, respectively. Then, 1 wt.% BDDGE was added to the original collagen solution as a cross-linking agent. The formation of nonwoven mats was carried out on a custom-built experimental setup for electrospinning (ELS) in which 1 mL of the polymer solution in HFIP was poured into a 10-mL plastic syringe and ejected through a polyethylene tube into a blunt needle (0.36 mm in diameter) at a flow rate of 2 mL/h using a Single Syringe Infusion Pump BYZ-810 (Hunan Beyond Medical Technology Co., Ltd., Changsha, China). The needle tip and a collector (a 7.5 cm × 9 cm sheet of aluminum foil) were connected to a high-voltage power supply set at 20 kV. The distance between the needle and the collector was 12 cm.

The electrospun collagen scaffolds were placed into a 15 wt.% BDDGE isopropanol solution at a temperature of 37 °C for 6 days to increase their mechanical properties. The pH level was maintained at 5.9 for the entire period. After the electrospinning, all the ELS PLGA mats were collected from the collector and kept at room temperature for 2 days to remove and evaporate any residual HFIP.

### 2.4. Extrusion 3D Printing with Simultaneous Photocuring

The initial photopolymerizable composition (PPC) consisted of an aqueous solution of 20 wt.% HAGM, 5 wt.% PEGDA, 0.1 wt.% flavin mononucleotide as a photoinitiator, and 0.5 wt.% triethanolamine. For the fabrication of scaffolds based on hyaluronic acid (3D HAGM), we used an original three-dimensional extrusion printer of our own design [38]. The process was based on a layer-by-layer application of PPC along a trajectory determined by a custom-written 3D computer model using G-code scripts, with simultaneous photocuring by laser radiation (λ = 445 nm). The PPC was poured into a 1-mL glass syringe and ejected through a blunt needle (0.15 mm inner diameter) onto the glass substrate placed on the printing platform. The PPC was extruded by pressing the syringe’s plunger at a flow rate of 1 µL/s with an average printing speed of 1 mm/s, with simultaneous photocuring by exposure to blue radiation (with light intensity up to 20 mW/cm^2^ at λ = 445 nm) from two focused laser sources. The fabricated 3D HAGM scaffolds (5 mm × 5 mm, 0.5 mm height, 5 layers, ~130 μm fiber diameter) were additionally cured under laser irradiation at a wavelength of 445 nm with a power of 1.5 W for 30 min.

### 2.5. Antisolvent 3D Printing

A PLGA solution for antisolvent 3D printing was prepared by dissolving the polymer in tetraglycol to a concentration of 10 wt.%. The formation of three-dimensional PLGA scaffolds was carried out using the antisolvent 3D printing technique proposed and developed by us earlier [39]. Desired structures were obtained through controlled extrusion according to the 3D computer model (6 mm diameter, 0.5 mm height, 85% filling density, and ~180 µm layer thickness). The PLGA solution was poured through a blunt needle (0.21 mm in diameter) at a flow rate of 0.05 µL/s onto the bottom of a Petri dish filled with distilled water at an average printing speed of 1 mm/s. After printing, the scaffolds were immersed in distilled water at 25 °C for 24 h to ensure final curing.

### 2.6. Microscopy

The microstructure and surface morphology of the experimental samples were studied using Phenom ProX (Phenom, Eindhoven, The Netherlands) and Scios (Thermo Fisher Scientific, Waltham, MA, USA) scanning electron microscopes using a secondary electron detector (Everhart-Thornley detector) in the Optiplan mode. The sizes and morphology of the UCNPs were determined using a Tecnai G212 SPIRIT transmission electron microscope (Thermo Fisher Scientific, Waltham, MA, USA). A quantitative assessment of the size distribution of the nanoparticles was carried out using the ImageJ program [40].

### 2.7. Analysis of Photoluminescent Properties of Polymer Scaffolds

The photoluminescence spectra of the studied samples of polymer scaffolds impregnated with UCNPs, when excited by continuous radiation of a semiconductor laser (JSC Semiconductor Devices, Saint Petersburg, Russia) with a wavelength of 976 nm, were recorded using a Fluorolog-3 spectrofluorimeter (Horiba Jobin Yvon, Longjumeau, France).

### 2.8. In Vivo Mice Imaging Study

The assessment of tissue response to the formed polymer scaffolds was carried out using the model of subcutaneous implantation in female mice of the BALB/c line (n = 12) aged 8–10 weeks, weighing 20–22 g. To ensure anesthesia, all animals were injected intramuscularly 5 min before surgical procedures with a combination of Zoletil at a dose of 0.1 mL/kg (Virbac, Carros, France) and Xylazine at a dose of 1 mg/kg (Nita-Pharm, Saratov, Russia). After that, a subcutaneous pocket with a depth of 1 cm was formed and the test samples were implanted, followed by skin suturing with an interrupted suture. The mice were kept under the conventional conditions of the Department of Laboratory Animals N.N. Blokhin. All animals received extruded food and free access to water. On the day of the start of the experiment, all mice were weighed and divided into groups of 3 each.

The photoluminescence parameters of the implanted scaffolds were analyzed using the imaging system described in [41] on the first, 4th, 7th, 11th, and 14th days of the experiment. An LDD-10 semiconductor laser (JSC Semiconductor Devices, Saint Petersburg, Russia) with a fiber output was used to generate excitation radiation at a wavelength of 976 nm. The laser beam was focused using a Raylase scanner head (Raylase, Wessling, Germany) with a radiation intensity of 200 mW/cm^2^. Detection of the UCNP photoluminescent signal was performed using a highly sensitive Falcon EMCCD camera (Raptor Photonics Incorporated, Larne, UK) equipped with an F = 0.95 objective. A system of interference filters (Semrock, Rochester, NY, USA) was used to notch the exciting laser radiation (976 nm). A quantitative assessment of the photoluminescence intensity of nanoparticles encapsulated in scaffolds was carried out using the ImageJ program.

All experiments with animals were carried out according to the ethical standards for the treatment of animals adopted by the local ethics committee for animal trials of the N.N. Blokhin Institution National Medical Research Center of Oncology of the Russian Ministry of Health, and the Geneva Convention on “International Guiding Principles for Biomedical Research Involving Animals” (Geneva, 1990).

### 2.9. Histological Assays

After animal euthanasia by CO_2_ inhalation on the 14th day, tissue biopsies with implanted scaffolds were fixed with 10% formalin for 72 h and embedded in paraffin blocks, and 5–10 µm histological sections were made using a HistoCore Arcadia C microtome (Leica, Wetzlar, Germany) according to the standard procedure [42]. The sections were then stained with hematoxylin and eosin according to the standard protocol. Images were captured using light microscopy (Zeiss Axio Observer.D1, Carl Zeiss Microscopy GmbH, Oberkochen, Germany) and tissue response was assessed in several fields of view (at least five for each sample). Inflammatory processes, the degree of bioresorption, and angiogenesis were assessed by counting the number of leukocytes per 1 mm^2^ of tissue, foreign-body giant cells per 1 mm^2^ of the scaffold, and the vascularized area, respectively.

### 2.10. Statistical Analysis

All data were presented as μ ± SD (standard deviation). Statistical analysis and graphing were performed with SigmaPlot v14.0 (Systat Software Inc., Palo Alto, Santa Clara, CA, USA). The differences between groups were assessed by one-way ANOVA using Tukey post hoc tests. Statistical significance was accepted for *p* < 0.05.

## 3. Results and Discussion

### 3.1. UCPNs

**Figure 1:**
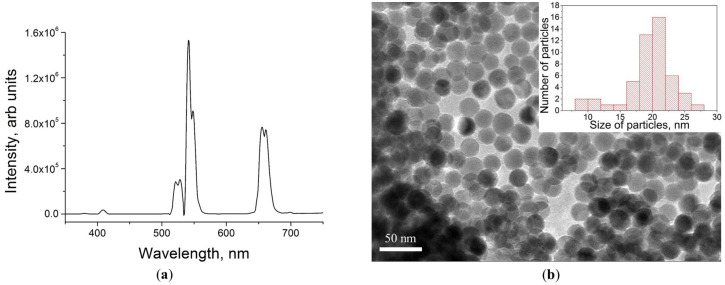
Characterization of β-NaYF_4_:Yb^3+^, Er^3+^/NaYF_4_ nanoparticles: (**a**) photoluminescence spectrum under 975 nm laser excitation (at intensity 5 W/cm^2^); (**b**) TEM image with size distribution histogram.

Upconversion nanoparticles of β-NaYF_4_:Yb^3+^, Er^3+^ with a NaYF_4_ inert shell were synthesized via the thermal decomposition of fluoride precursors. This method makes it possible to synthesize particles with a sufficient degree of monodispersity (Figure 1b), which is crucial for the photoluminescent analysis of the scaffold material impregnated with nanoparticles.

We characterized the photoluminescent properties of the UCNPs. Figure 1 shows the photoluminescence spectrum, TEM image, and size distribution histogram of UCNPs with a core/shell structure and an average diameter of 21 ± 6 nm. Upconversion nanoparticles doped with Er^3+^ ions are characterized by two main photoluminescence bands at wavelengths of 544 and 658 nm under 975 nm excitation [37]. UCNP photoluminescence under NIR-light irradiation is the result of energy conversion in pairs of Yb^3+^ and Er^3+^ ions. The Yb^3+^ absorbs 980-nm light and passes into an excited metastable state, then non-radiatively transfers energy to neighboring Er^3+^, which in turn emits light due to transitions from ^2^H_11/2_/^4^S_3/2_ and ^4^F_9/2_ excited states to the ^4^I_15/2_ ground state [43]. Due to the noticeable difference in intensity of photoluminescence bands at wavelengths of 544 and 658 nm, UCNPs can serve not only for visualization but also for local temperature measurement [44].

### 3.2. UCNP-Loaded Polymer Scaffolds

We produced four types of UCNP-loaded scaffolds, including ELS COL, ELS PLGA, 3D PLGA, and 3D HAGM, in concentrations of 1 mg of UCNPs per 100 mg of polymer (1%). The UCNPs were added to the initial compositions for electrospinning and 3D printing. For uniform distribution of nanoparticles in the solution and, as a result, in the scaffold, all initial solutions were sonicated in an ultrasound bath for 60 min.

The electrospinning method allows the creation of highly porous nonwoven structures imitating an intercellular matrix. This can be achieved due to the chaotic deflection of the polymer solution jets in an electric field. A Taylor cone was formed at the end of the needle by applying an electric field to a polymer solution in a readily volatile polar solvent. From this cone, both a spray (at a low viscosity of the solution) and jets can be formed. By changing the needle—collector voltage and the solution viscosity, it was possible to achieve the desired degree of porosity and fiber thickness and avoid the formation of spray drops [45]. Before the formation of the UCNP-loaded scaffold, several experiments were performed to select suitable electrospinning parameters for preparing bead-free nanofibers. Moreover, the technology of this method provided additional uniformity of the nanoparticle distribution, since all fibers were randomly deposited on the collector.

The electrospun collagen mats were immersed in a 15 wt.% BDDGE isopropanol solution for six days for cross-linking and stabilization. Without this procedure, the collagen mats can lose their structural integrity and mechanical properties due to dissolution in HFIP and exposure to an electric field [46]. Due to the chosen cross-linking method, the samples retained their fibrous structure for several days in the aqueous solutions, ensuring cell adhesion [20].

3D printing is a method of rapid scaffold prototyping that enables a strictly defined shape with less material consumption. After selecting the printing parameters (solution viscosity, flow rate, and printing head speed), it is possible to design and build a complicated structure with the required porosity and resolution. Printing resolution is one of the most important parameters of this method. Since fairly large amounts of liquid solution are required for printing, additional stabilization is necessary for the sample formation printing resolution intended for this purpose.

This approach of extrusion 3D printing with simultaneous photocuring enables precise control over the properties of HA derivatives, depending on the end goal for their use [36]. The process of photo-initiated crosslinking was realized by using a mononucleotide/triethanolamine complex characterized by an absorption band around 450 nm to produce free radicals [47]. The 3D HAGM scaffolds were stabilized under laser irradiation at a wavelength of 445 nm at a power of 1.5 W for 30 min. The 3D PLGA scaffolds were immersed in distilled water at 25 °C for 24 h to remove any traces of tetraglycol and ensure final curing. The selected antisolvent and extrusion printing methods do not use toxic solvents and high temperatures, which allows them to be applied in various areas of tissue engineering.

These two fundamentally different methods have advantages and disadvantages, and can be used to solve various tissue engineering problems [48]. A soft structure with a large surface area can be applied in targeted therapy and localized drug delivery [49], whereas a rigid scaffold with a well-defined shape is more suitable for repairing defects, for example, in cartilage and bone tissue [50].

**Figure 2:**
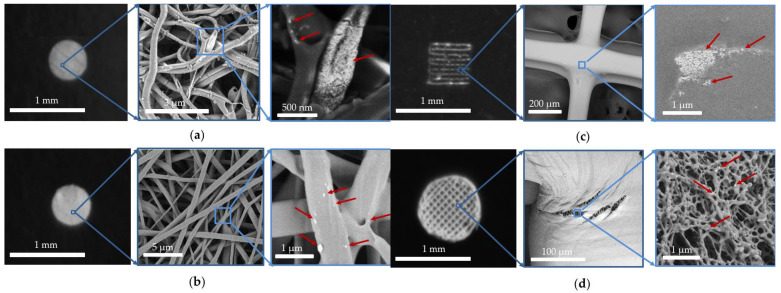
Photographs and SEM images of the scaffold surface: (**a**) ELS COL, (**b**) ELS PLGA, (**c**) 3D PLGA, (**d**) 3D HAGM. The red arrows show the UCNPs embedded in the scaffold material.

**Figure 3:**
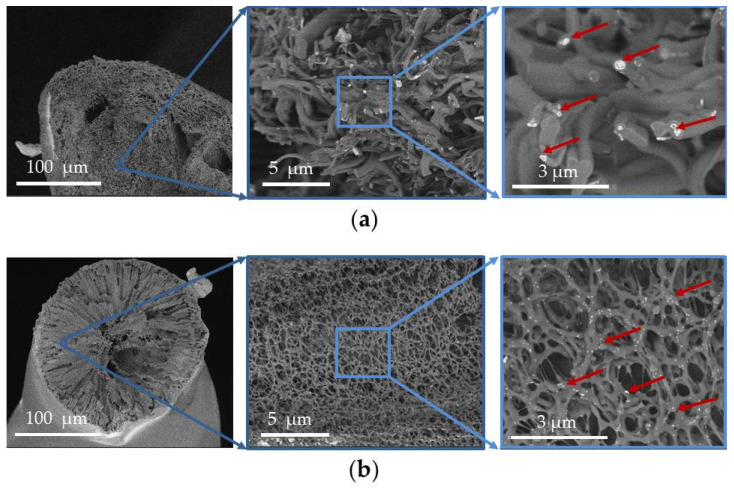
SEM images of the transverse sections of the scaffolds: (**a**) ELS COL, (**b**) 3D PLGA. The red arrows show the UCNPs embedded in the scaffold material.

Figure 2 shows SEM images of the surfaces of the designed scaffold. The UCNPs were mainly located inside the scaffold fibers on chips, cuts, or pores. There were normally none on the surfaces. A SEM study of transverse sections of the scaffolds (Figure 3) confirmed that the UCNPs were distributed uniformly and isolated from the external environment by the polymer material. Consequently, the nanoparticles loaded into the scaffold are isolated from the external environment for the most part. This was also confirmed by our previous results relating to the release of UCNPs from a scaffold into an aqueous medium, described in [20]. It was shown that the photoluminescence spectrum of UCNPs released into the aqueous medium has a higher ratio of the intensity of the red band at the wavelength of 658 nm to the intensity of the green band at a wavelength of 544 nm, compared to the same intensity ratio for UCNPs inside the scaffold. The intensity of the red photoluminescence band increased with both an increase in the power density of the exciting radiation and with certain changes in environmental conditions, but the mechanisms are different. An increase in radiation power density leads to a tree-quantum process of population of excited states (^4^G_11/2_ and ^4^G_7/2_), from which a nonradiative relaxation to the ^4^F_9/2_ excited state is more probable than to the ^2^H_11/2_/^4^S_3/2_ excited states [43]. In turn, the influence of the environment can lead to quenching and (in our case, because of the presence of H_2_O molecules) a forced transition from a metastable 4I_13/2_ state to a ^4^F_9/2_ excited state [51].

Isolation of the nanoparticles’ surfaces from the external environment can enable better imaging, since the intensity of photoluminescence of nanoparticles inside the body will decrease much less. Nanoparticles coated with a polymer layer were less susceptible to quenching, since the influence of H_2_O molecules was excluded.

In addition, we carried out an MTT assay using Bj-5ta fibroblasts, which showed that none of the above scaffolds were cytotoxic and provided acceptable conditions for primary cell adhesion, proliferation, and colonization [20].

### 3.3. In Vivo Mice Imaging Study

**Figure 4:**
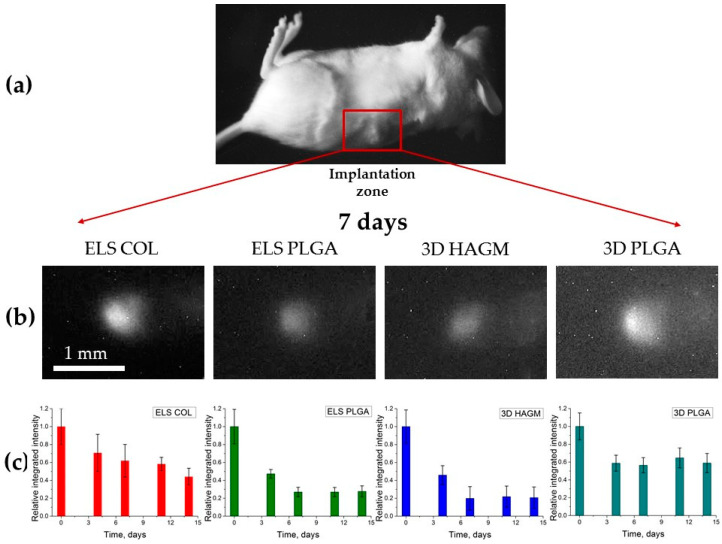
Bioimaging of scaffolds: (**a**) bright-field photo of animal showing implantation zone; (**b**) photoluminescent images of scaffolds detected under mouse skin at 975-nm laser excitation (7th day after scaffolds implantation); (**c**) histograms indicating the decrease of photoluminescent signal during the in vivo experiment.

**Figure A1:**
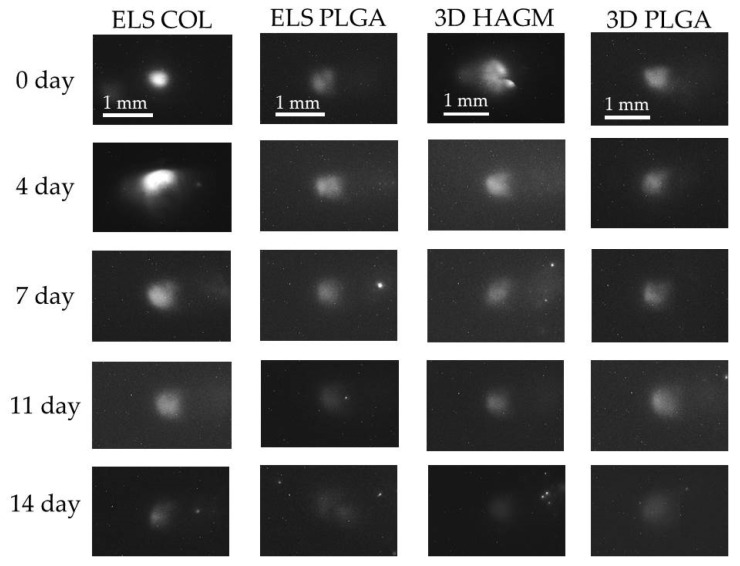
Bioimaging of scaffolds. Photoluminescent images of scaffolds detected under the mice skin at 975-nm laser excitation on the first, 4th, 7th, 11th, and 14th days of the experiment.

The photoluminescent signal from the polymeric scaffolds was evaluated during the 14 days after their subcutaneous implantation in female BALB/c mice (Figure 4). It was found that the photoluminescence intensity of all samples monotonically decreased throughout the in vivo experiment (Figure A1). This decrease was most noticeable from the first to the seventh day of the experiment, indicating a gradual degradation of the scaffolds in the animal tissues. On the fourth day of the experiment, erosion of the photoluminescence area was detected, indicating scaffold biodegradation accompanied by the release of UCNPs into the surrounding tissues.

The contribution of the red photoluminescence band at the wavelength of 658 nm to UCNP integrated intensity was found using a system of optical filters. The intensity was 85% of the total value, and there were no significant differences among the scaffolds.

In general, the photoluminescence intensity of UCNPs encapsulated in the implanted scaffolds decreased with time. At the same time, the ELS COL and 3D PLGA scaffolds seemed to be the most stable, since the photoluminescence intensity of the encapsulated nanoparticles decreased more slowly than in the other samples. The ELS PLGA and 3D HAGM samples were the least stable, because the photoluminescence intensity decreased sharply after the first week of the experiment.

### 3.4. In Vivo Biocompatibility Assessment

**Figure 5:**
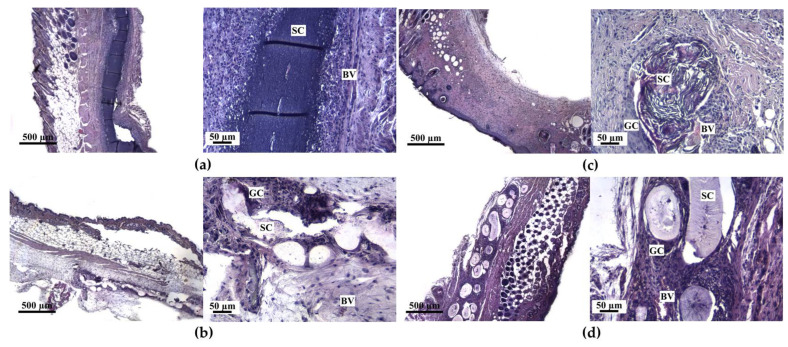
Histological images of the tissue with scaffold sections stained with hematoxylin and eosin, excised 14 days after implantation: (**a**) ELS COL, (**b**) ELS PLGA, (**c**) 3D PLGA, (**d**) 3D HAGM. SC—scaffold material, BV—blood vessel, GC—foreign-body giant cells.

Fourteen days after the scaffold implantation the mice were sacrificed for histological analysis (see Figure 5). The scaffold material was detected in every stained section, and the tissue from the immediate vicinity of the implant was examined. The groups were characterized by the presence of young granulation tissue with full blood vessels and inflammatory cells in the implantation zone (see Figure 5).

**Figure 6:**
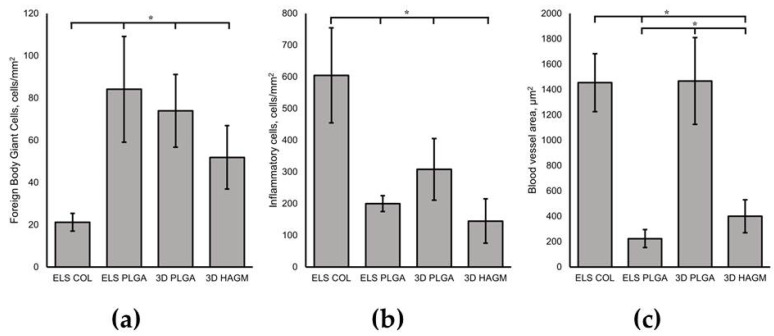
Biocompatibility in vivo: (**a**) foreign-body giant cells per 1 mm^2^; (**b**) inflammatory cells per 1 mm^2^; (**c**) vascularized area, * *p* < 0.05.

The collagen scaffolds caused a moderate inflammatory response, characterized by the presence of a large number of segmented leukocytes in the surrounding tissue. Blood vessels were concentrated on the periphery of the ELS COL scaffold, contributing to increased leukocyte infiltration, which, in turn, inhibited material resorption processes and could cause a decrease in the photoluminescence intensity of the initially embedded UCNPs. When studying tissue fragments with implanted polylactide-glycolic acid scaffolds (both electrospun and 3D-printed) a 2-fold decrease in the number of inflammatory cells compared to ELS COL was observed, proving that PLGA scaffold fabrication techniques ensured implant biocompatibility in vivo. This may be caused by residual crosslinking agents in the collagen samples. The ELS PLGA scaffolds were surrounded by foreign-body giant cells (a 4-fold increase vs. ELS COL) and underwent an intense biodegradation process (see Figure 6a). There were no statistically significant differences in immune response between the 3D and ELS PLGA scaffolds. This can be explained by their identical chemical composition. However, the difference in the shape of these two types of scaffolds affected another parameter. The 3D-printed porous PLGA scaffolds facilitated angiogenesis and promoted more efficient vessel invasion into the connective tissue thickness in contrast to electrospun ones (a 7-fold increase vs. ELS PLGA, see Figure 6c). Moreover, active cell infiltration into the 3D printed fiber micropores was observed, contributing to its exceptional biocompatibility. Examination of the biopsy specimens also revealed that the ELS PLGA and ELS COL scaffolds decreased in diameter from 4 to 2 mm and the 3D PLGA scaffolds decreased in diameter from 6 mm to 3 mm. The HAGM-based scaffolds were almost completely biodegraded on the 14th day after implantation, and apparently had no toxic effects (less than 200 cells per 1 mm^2^ of tissue) on the tissue, as it was characterized by an insignificant number of inflammatory cells (see Figure 6b).

The results shown in Figure 4 and Figure 5 show the correlation between the photoluminescence and histological analyses. The ELS COL and 3D PLGA scaffolds retained their structure (Figure 5a,c), as confirmed by a 50% decrease in photoluminescence intensity on day 14 of the experiment. Only the ELS PLGA and 3D HAGM scaffold fragments were detected in the histological analysis. Their photoluminescence intensity was about 50% lower than that of the ELS COL and 3D PLGA samples on the last day of the experiment.

## 4. Conclusions

In this study, we synthesized UCNP-loaded scaffolds based on synthetic (PLGA) and natural (COL, HAGM) polymers using electrospinning and 3D printing methods. We showed that without any surface modifications, the upconversion nanoparticles could be included in the initial polymer solution for scaffold formation. A concentration of 1 wt.% of UCNPs in the initial solution enabled uniform material photoluminescence with excitation at a wavelength of 975 nm. We proved the possibility of UCNP-loaded scaffold bioimaging for at least 14 days. Scaffold biocompatibility was analyzed using a subcutaneous implantation model in female BALB/c mice. The inflammatory response of the tissues surrounding the implanted scaffolds was moderate. The HAGM- and PLGA-based scaffolds were the most biocompatible of the synthesized samples. The 3D-printed PLGA scaffolds promoted the most efficient vessel invasion into the connective tissue thickness. Monitoring the photoluminescent signal from the scaffold material allowed us to prove the correlation between the photoluminescent signal from UCNP and the scaffold degradation rate. Thus, we demonstrated the possibilities of UCNP-assisted luminescent bioimaging for continuously monitoring scaffold degradation in vivo.

UCNP-assisted luminescent bioimaging is now actively developing, and there are still unanswered questions. It is important to investigate how UCNPs influence scaffold properties such as mechanical characteristics, cell adhesion and cytokine response. Study of the long-term toxicity of UCNP-loaded scaffolds is our main goal for further research.

## Data Availability

Available upon request.
